# Supplementary material for: Impact of LS Mutation on Pharmacokinetics of Preventive HIV Broadly Neutralizing Monoclonal Antibodies: A Cross-Protocol Analysis of 16 Clinical Trials in People without HIV
Source: Pharmaceutics. 2024 Apr 27;16(5):594. doi: 10.3390/pharmaceutics16050594 (PMC11125931; doi:10.3390/pharmaceutics16050594)
Supplement: Supplementary file 1 [file pharmaceutics-16-00594-s001.zip › pharmaceutics-2985042-Supplemental Materials.html]

PK model diagnostic Plots for combined study


# PK model diagnostic Plots for combined study

## PK model diagnostic Plots for combined study

- 1 Model diagnostic plot list:
- 2 VRC01
- 3 VRC01LS
- 4 VRC07.523LS
- 5 PGT121
- 6 PGT121LS
- 7 PGDM1400
- 8 PGDM1400LS
- 9 3BNC117
- 10 3BNC117LS
- 11 10-1074
- 12 10-1074LS

Lily Zhang

29 December, 2023

# 1 Model diagnostic plot list:

- Observations vs. population predicted value (PRED) in both linear and log scales with a line of identity and a regression line
- Observations vs. individual PRED in both linear and log scales with a line of identity and a regression line
- Individual WRES vs. individual PRED
- Individual WRES vs. time or time after dose
- Histogram and/or QQ plot of IWRES
- Assessment of shrinkage of ETA
- Observations (or dependent variable), individual PRED, and PRED concentrations vs. time (overlaid and/or side by side)
- VPCs

Byon et al. 2013: “Establishing Best Practices and Guidance in Population Modeling: An Experience With an Internal Population Pharmacokinetic Analysis Guidance”

# 2 VRC01

Figure 2.1: Observations vs Predictions; scatterplot of the residuals; QQ plot of the residuals; distribution of the random effects

Figure 2.2: Individual fits

Figure 2.3: Individual fits

Figure 2.4: Individual fits

Figure 2.5: Individual fits

Figure 2.6: Individual fits

Figure 2.7: Individual fits

Figure 2.8: Individual fits

Figure 2.9: Individual fits

Figure 2.10: Individual fits

Figure 2.11: Individual fits

Figure 2.12: Individual fits

Figure 2.13: Individual fits

Figure 2.14: VPC

# 3 VRC01LS

Figure 3.1: Observations vs Predictions; scatterplot of the residuals; QQ plot of the residuals; distribution of the random effects

Figure 3.2: Individual fits

Figure 3.3: Individual fits

Figure 3.4: Individual fits

Figure 3.5: Individual fits

Figure 3.6: VPC

# 4 VRC07.523LS

Figure 4.1: Observations vs Predictions; scatterplot of the residuals; QQ plot of the residuals; distribution of the random effects

Figure 4.2: Individual fits

Figure 4.3: Individual fits

Figure 4.4: Individual fits

Figure 4.5: Individual fits

Figure 4.6: Individual fits

Figure 4.7: Individual fits

Figure 4.8: Individual fits

Figure 4.9: Individual fits

Figure 4.10: Individual fits

Figure 4.11: Individual fits

Figure 4.12: Individual fits

Figure 4.13: Individual fits

Figure 4.14: VPC

# 5 PGT121

Figure 5.1: Observations vs Predictions; scatterplot of the residuals; QQ plot of the residuals; distribution of the random effects

Figure 5.2: Individual fits

Figure 5.3: Individual fits

Figure 5.4: Individual fits

Figure 5.5: VPC

# 6 PGT121LS

Figure 6.1: Observations vs Predictions; scatterplot of the residuals; QQ plot of the residuals; distribution of the random effects

Figure 6.2: Individual fits

Figure 6.3: Individual fits

Figure 6.4: Individual fits

Figure 6.5: VPC

# 7 PGDM1400

Figure 7.1: Observations vs Predictions; scatterplot of the residuals; QQ plot of the residuals; distribution of the random effects

Figure 7.2: Individual fits

Figure 7.3: Individual fits

Figure 7.4: Individual fits

Figure 7.5: VPC

# 8 PGDM1400LS

Figure 8.1: Observations vs Predictions; scatterplot of the residuals; QQ plot of the residuals; distribution of the random effects

Figure 8.2: Individual fits

Figure 8.3: VPC

# 9 3BNC117

Figure 9.1: Observations vs Predictions; scatterplot of the residuals; QQ plot of the residuals; distribution of the random effects

Figure 9.2: Individual fits

Figure 9.3: Individual fits

Figure 9.4: Individual fits

Figure 9.5: VPC

# 10 3BNC117LS

Figure 10.1: Observations vs Predictions; scatterplot of the residuals; QQ plot of the residuals; distribution of the random effects

Figure 10.2: Individual fits

Figure 10.3: Individual fits

Figure 10.4: Individual fits

Figure 10.5: Individual fits

Figure 10.6: VPC

# 11 10-1074

Figure 11.1: Observations vs Predictions; scatterplot of the residuals; QQ plot of the residuals; distribution of the random effects

Figure 11.2: Individual fits

Figure 11.3: Individual fits

Figure 11.4: Individual fits

Figure 11.5: VPC

# 12 10-1074LS

Figure 12.1: Observations vs Predictions; scatterplot of the residuals; QQ plot of the residuals; distribution of the random effects

Figure 12.2: Individual fits

Figure 12.3: Individual fits

Figure 12.4: Individual fits

Figure 12.5: Individual fits

Figure 12.6: VPC
