# Supplementary material for: Impact of LS Mutation on Pharmacokinetics of Preventive HIV Broadly Neutralizing Monoclonal Antibodies: A Cross-Protocol Analysis of 16 Clinical Trials in People without HIV
Source: Pharmaceutics. 2024 Apr 27;16(5):594. doi: 10.3390/pharmaceutics16050594 (PMC11125931; doi:10.3390/pharmaceutics16050594)

Supplemental Materials for: Impact of LS mutation on pharmacokinetics of preventive HIV broadly neutralizing monoclonal antibodies: a cross-protocol analysis of 16 clinical trials in people without HIV

Table S1: List of mAbs and descriptions

mAb	HIV-1 epitope specificity	Description
**VRC01**	**CD4 binding site**	A potent and broadly neutralizing HIV-1 human mAb targeted against the HIV-1 CD4 binding site. It was originally discovered in an individual infected with HIV-1 for more than 15 years and whose immune system controlled the virus without antiretroviral therapy(2,3). By applying a novel method of isolating B cells that produce a specific antibody, and using recombinant DNA technology, the heavy and light chains encoding VRC01 were cloned and sequenced, allowing the synthetic production of codon-optimized genes encoding the variable region that was inserted into proprietary IgG1 background sequences (2).
**VRC01LS**		An engineered variant of VRC01 that uses the same IgG1 background sequences as VRC01. The LS designation specifies methionine to leucine (L) and asparagine to serine (S) (M428L/N434S, referred to as LS) changes within the C-terminus of the heavy chain constant region. The LS mutation was introduced by site-directed mutagenesis to increase the binding affinity for the neonatal Fc-receptor (FcRn), resulting in increased recirculation of functional IgG (14, 17) and thereby increasing plasma half-life.
**VRC07-523LS**		An engineered variant related to VRC01. The VRC07 (parental) heavy chain was identified by deep sequencing of heavy chain transcripts from peripheral blood B cells isolated also from donor 45 (2).based on its similarity to the VRC01 mAb and paired with the VRC01 (parental) light chain. "07" denotes sequential numbering when discovered. To increase neutralization potency and breadth, a series of amino acid mutations were introduced; mutations that resulted in autoreactivity were rejected(2).The mutations that define the 523 designation are a glycine to histidine mutation at residue 54 of the heavy chain, a deletion of the first two amino acids, glutamate and isoleucine, from the light chain, and a valine to serine mutation at the third amino acid residue of the light chain.
**3BNC117**		A potent and broadly neutralizing HIV-1 mAb that specifically binds to the CD4 binding site within HIV-1 envelope gp120 {Caskey, 2015 #70}. It was isolated from a viremic controller (4).
**3BNC117-LS**		An engineered variant of 3BNC117 that uses the same IgG1-kappa background sequences as 3BNC117. The LS designation specifies M428L/N434S changes within the C-terminus of the heavy chain constant region far outside of the antigen-combining site. This version of 3BNC117-LS also has a glycan tuning modification.
**PGDM1400**	**V1V2 Glycan**	A potent and broadly neutralizing HIV-1 mAb targeted against the glycans in the region of N160 on the V2 loop of gp120 Env. It was identified from African donor 84 of the IAVI Protocol G cohort (13).
**PGDM1400LS**		An engineered variant of PGDM1400 that uses the same IgG1 background sequences as PGDM1400. The LS designation specifies M428L/N434S changes within the C-terminus of the heavy chain constant region far outside of the antigen-combining site.
**PGT121**	**V3 Glycan**	A potent and broadly neutralizing HIV-1 mAb that targets the V3 glycan-dependent epitope region of the HIV-1 virus. It was identified from African donor 17 of the IAVI Protocol G cohort. It targets the V3 glycan-dependent epitope region of the HIV-1 virus
**PGT121.414.LS**		An engineered variant of PGDM1400 that uses the same IgG1 background sequences as PGDM1400. It contains a total of 8 residue modifications to improve various aspects of manufacturing, stability, and in vivo elimination half-life. Six of the modifications are in the Fragment crystallizable (Fc) region, providing increased conformational stability leading to improved manufacturing characteristics including low pH stability and an improved storage stability profile. The 2 modifications in the Fc region of each heavy chain are the Xencor Xtend LS modifications helping provide a significantly reduced elimination half-life in vivo. The LS designation specifies M428L/N434S changes within the C-terminus of the heavy chain constant region far outside of the antigen-combining site.
**10-1074**		A potent and broadly neutralizing HIV-mAb that that specifically binds to the V3 loop within HIV-1 envelope gp120 (5,6). It was isolated from an individual who acquired a Clade A HIV-1 virus.
**10-1074-LS**		An engineered variant of 10-1074 that uses the same IgG1-lambda background sequences as 10-1074. The LS designation specifies M428L/N434S changes within the C-terminus of the heavy chain constant region far outside of the antigen-combining site.

Supplemental Table S2: **Lower limits of quantification (LLoQ) of each assay by monoclonal antibody and study**

mAb	Protocol	Assay	LLoQ (mcg/mL)
VRC01	HVTN104	ELISA	1.1
VRC01	VRC602	ELISA	1.1
3BNC117	YCO-0899	ELISA	0.316
3BNC117	YCO-0899	ELISA	0.633
3BNC117	MCA-0835	ELISA	0.25
3BNC117	MCA-0835	ELISA	0.48
3BNC117LS	YCO-0946	ELISA	0.635
3BNC117LS	YCO-0971	ELISA	0.635
10-1074	YCO-0899	ELISA	0.336
10-1074	MCA-885	ELISA	0.2
10-1074LS	YCO-0971	ELISA	0.12
PGT121	T002	BAMA	0.5
PTDM1400	T002	BAMA	0.24
VRC07-523LS	VRC605	ELISA	1.0
VRC07-523LS	HVTN127/HPTN087	BAMA	0.02285

Supplemental Table S3: Demographics of included study participants from each study

**Characteristic**	**VRC01** N = 169	**VRC01 LS** N = 56	**VRC07-523LS** N = 171	**3BNC117** N = 40	**3BNC117LS** N = 50	**PGDM1400** N = 33	**PGDM1400LS** N = 15	**PGT121** N = 40	**PGT121.414 LS** N = 33	**10-1074** N = 37	**10-1074LS** N = 56
Age (years), Median (IQR)	28 (24, 34)	30 (26, 36)	27 (24, 35)	44 (30, 52)	47 (36, 55)	26 (23, 32)	27 (26, 34)	25 (23, 28)	31 (26, 38)	41 (30, 52)	49 (35, 55)
Sex at Birth											
Female, n (%)	81 (48%)	28 (50%)	100 (58%)	8 (20%)	23 (46%)	16 (48%)	10 (67%)	24 (60%)	19 (58%)	8 (22%)	19 (34%)
Male, n (%)	88 (52%)	28 (50%)	71 (42%)	32 (80%)	27 (54%)	17 (52%)	5 (33%)	16 (40%)	14 (42%)	29 (78%)	37 (66%)
Weight (Kg), Median (IQR)	73 (63, 86)	71 (65, 88)	73 (63, 85)	81 (67, 89)	79 (70, 93)	71 (61, 84)	72 (65, 76)	71 (64, 83)	76 (67, 89)	82 (68, 88)	83 (71, 95)
Creatinine (mL/min), Median (IQR)	127 (110, 147)	125 (108, 149)	122 (107, 139)	110 (95, 133)	109 (88, 124)	120 (101, 147)	116 (102, 139)	124 (115, 147)	128 (107, 144)	108 (98, 126)	102 (88, 127)

Supplemental Table S4: Population pharmacokinetic parameter estimates from the base model without adjusting for covariates and with no correlation parameters based on trial-pooled models for each mAb. The same random effects were considered for the parental and LS data. CI, confidence interval; % RSE, % relative standard error (SE/Estimate * 100); SD, standard deviation of the random effect; SE, standard error of the error terms.

mAb	Parameter	Description	Estimate	95% CI	% RSE
VRC01	F	Bioavailability after SC administration	0.669	(0.617, 0.72)	3.92
	Ka (1/day)	Absorption rate constant	0.199	(0.152, 0.246)	12.09
	CL (L/day)	Clearance from the central compartment	0.345	(0.327, 0.364)	2.73
	Vc (L)	Volume of the central compartment	1.999	(1.851, 2.148)	3.78
	Q (L/day)	Inter-compartmental distribution clearance	0.835	(0.758, 0.912)	4.72
	Vp (L)	Volume of the peripheral compartment	4.12	(3.85, 4.391)	3.35
	ωCL	SD, inter-individual CL	0.341	(0.302, 0.379)	5.77
	ωVc	SD, inter-individual Vc	0.291	(0.222, 0.361)	12.18
	ωQ	SD, inter-individual Q	0.301	(0.208, 0.394)	15.75
	ωVp	SD, inter-individual Vp	0.373	(0.316, 0.431)	7.88
	σ (constant)	SE, additive error	0.472	(0.362, 0.583)	11.89
	σ (proportional)	SE, proportional error	0.218	(0.208, 0.228)	2.35
VRC01LS	F	Bioavailability after SC administration	0.6	(0.438, 0.761)	13.76
	Ka (1/day)	Absorption rate constant	0.232	(0.11, 0.353)	26.68
	CL (L/day)	Clearance from the central compartment	0.036	(0.031, 0.041)	6.83
	Vc (L)	Volume of the central compartment	1.072	(0.743, 1.4)	15.64
	Q (L/day)	Inter-compartmental distribution clearance	1.131	(0.275, 1.988)	38.63
	Vp (L)	Volume of the peripheral compartment	1.975	(1.655, 2.295)	8.26
	ωCL	SD, inter-individual CL	0.327	(0.255, 0.4)	11.34
	ωVc	SD, inter-individual Vc	0.697	(0.518, 0.875)	13.06
	ωQ	SD, inter-individual Q	1.309	(0.795, 1.824)	20.05
	ωVp	SD, inter-individual Vp	0.434	(0.319, 0.549)	13.53
	σ (proportional)	SE, proportional error	0.26	(0.245, 0.275)	2.88
VRC07-523LS	F	Bioavailability after SC administration	0.486	(0.454, 0.518)	3.35
	Ka (1/day)	Absorption rate constant	0.282	(0.245, 0.318)	6.7
	CL (L/day)	Clearance from the central compartment	0.115	(0.109, 0.121)	2.65
	Vc (L)	Volume of the central compartment	2.996	(2.702, 3.29)	5.01
	Q (L/day)	Inter-compartmental distribution clearance	0.405	(0.347, 0.464)	7.37
	Vp (L)	Volume of the peripheral compartment	3.894	(3.591, 4.197)	3.96
	ωCL	SD, inter-individual CL	0.302	(0.266, 0.338)	6.05
	ωVc	SD, inter-individual Vc	0.497	(0.436, 0.559)	6.36
	ωQ	SD, inter-individual Q	0.61	(0.496, 0.725)	9.58
	ωVp	SD, inter-individual Vp	0.385	(0.324, 0.446)	8.04
	σ (constant)	SE, additive error	0.135	(0.096, 0.173)	14.77
	σ (proportional)	SE, proportional error	0.174	(0.166, 0.182)	2.3
3BNC117	CL (L/day)	Clearance from the central compartment	0.688	(0.598, 0.779)	6.7
	Vc (L)	Volume of the central compartment	4.263	(3.719, 4.808)	6.51
	Q (L/day)	Inter-compartmental distribution clearance	2.279	(1.806, 2.753)	10.6
	Vp (L)	Volume of the peripheral compartment	9.561	(8.664, 10.458)	4.79
	ωCL	SD, inter-individual CL	0.418	(0.323, 0.512)	11.56
	ωVc	SD, inter-individual Vc	0.394	(0.303, 0.485)	11.81
	ωQ	SD, inter-individual Q	0.461	(0.25, 0.672)	23.37
	ωVp	SD, inter-individual Vp	0.256	(0.179, 0.334)	15.47
	σ (constant)	SE, additive error	0.109	(0.053, 0.166)	26.3
	σ (proportional)	SE, proportional error	0.219	(0.205, 0.234)	3.4
3BNC117-LS	F	Bioavailability after SC administration	0.587	(0.562, 0.613)	2.2
	Ka (1/day)	Absorption rate constant	0.406	(0.36, 0.453)	5.87
	CL (L/day)	Clearance from the central compartment	0.07	(0.064, 0.077)	4.67
	Vc (L)	Volume of the central compartment	3.025	(2.582, 3.467)	7.46
	Q (L/day)	Inter-compartmental distribution clearance	0.181	(0.137, 0.225)	12.43
	Vp (L)	Volume of the peripheral compartment	2.785	(2.41, 3.161)	6.88
	ωCL	SD, inter-individual CL	0.306	(0.242, 0.369)	10.66
	ωVc	SD, inter-individual Vc	0.438	(0.344, 0.531)	10.93
	ωQ	SD, inter-individual Q	0.544	(0.322, 0.765)	20.77
	ωVp	SD, inter-individual Vp	0.368	(0.26, 0.475)	14.92
	σ (proportional)	SE, proportional error	0.195	(0.185, 0.205)	2.59
PGDM1400	CL (L/day)	Clearance from the central compartment	0.207	(0.194, 0.221)	3.33
	Vc (L)	Volume of the central compartment	3.247	(2.797, 3.697)	7.07
	Q (L/day)	Inter-compartmental distribution clearance	0.212	(0.136, 0.288)	18.35
	Vp (L)	Volume of the peripheral compartment	1.849	(1.592, 2.105)	7.08
	ωCL	SD, inter-individual CL	0.186	(0.138, 0.234)	13.24
	ωVc	SD, inter-individual Vc	0.377	(0.278, 0.476)	13.43
	ωQ	SD, inter-individual Q	0.812	(0.57, 1.054)	15.2
	ωVp	SD, inter-individual Vp	0.281	(0.184, 0.378)	17.66
	σ (constant)	SE, additive error	0.129	(0.061, 0.197)	27.03
	σ (proportional)	SE, proportional error	0.117	(0.103, 0.13)	5.9
PGDM1400LS	F	Bioavailability after SC administration	0.648	(0.405, 0.891)	19.11
	Ka (1/day)	Absorption rate constant	0.484	(0.344, 0.624)	14.77

	CL (L/day)	Clearance from the central compartment	0.051	(0.041, 0.06)	9.44
	Vc (L)	Volume of the central compartment	3.59	(2.698, 4.481)	12.67
	Q (L/day)	Inter-compartmental distribution clearance	0.032	(-0.023, 0.087)	86.69
	Vp (L)	Volume of the peripheral compartment	0.387	(0.085, 0.689)	39.86
	ωCL	SD, inter-individual CL	0.186	(0.101, 0.27)	23.15
	ωVc	SD, inter-individual Vc	0.288	(0.176, 0.399)	19.72
	σ (constant)	SE, additive error	5.862	(3.454, 8.269)	20.96
	σ (proportional)	SE, proportional error	0.083	(0.059, 0.106)	14.62
PGT121	F	Bioavailability after SC administration	0.387	(0.196, 0.577)	25.18
	Ka (1/day)	Absorption rate constant	0.384	(0.2, 0.568)	24.46
	CL (L/day)	Clearance from the central compartment	0.305	(0.276, 0.333)	4.73
	Vc (L)	Volume of the central compartment	3.998	(3.448, 4.548)	7.02
	Q (L/day)	Inter-compartmental distribution clearance	0.735	(0.578, 0.893)	10.9
	Vp(L)	Volume of the peripheral compartment	5.039	(4.604, 5.474)	4.41
	ωCL	SD, inter-individual CL	0.246	(0.19, 0.302)	11.6
	ωVc	SD, inter-individual Vc	0.297	(0.222, 0.372)	12.87
	ωQ	SD, inter-individual Q	0.593	(0.413, 0.773)	15.47
	ωVp	SD, inter-individual Vp	0.232	(0.169, 0.295)	13.86
	σ (constant)	SE, additive error	0.222	(0.176, 0.269)	10.69
	σ (proportional)	SE, proportional error	0.096	(0.087, 0.105)	4.72
PGT121.414.LS	F	Bioavailability after SC administration	0.783	(0.647, 0.918)	8.84
	Ka (1/day)	Absorption rate constant	0.277	(0.233, 0.321)	8.14
	CL (L/day)	Clearance from the central compartment	0.062	(0.054, 0.069)	6.2
	Vc (L)	Volume of the central compartment	2.942	(2.363, 3.52)	10.03
	Q (L/day)	Inter-compartmental distribution clearance	0.541	(0.419, 0.663)	11.47
	Vp (L)	Volume of the peripheral compartment	3.023	(2.617, 3.428)	6.85
	ωCL	SD, inter-individual CL	0.293	(0.216, 0.369)	13.33
	ωVc	SD, inter-individual Vc	0.509	(0.37, 0.647)	13.86
	ωQ	SD, inter-individual Q	0.454	(0.271, 0.638)	20.59
	ωVp	SD, inter-individual Vp	0.304	(0.208, 0.4)	16.12
	σ (constant))	SE, additive error	1.104	(0.77, 1.439)	15.46
	σ (proportional)	SE, proportional error	0.076	(0.067, 0.085)	6.26
10-1074	CL (L/day)	Clearance from the central compartment	0.165	(0.139, 0.19)	7.92
	Vc (L)	Volume of the central compartment	2.576	(2.112, 3.041)	9.2
	Q (L/day)	Inter-compartmental distribution clearance	0.426	(0.306, 0.545)	14.3
	Vp (L)	Volume of the peripheral compartment	2.34	(2.016, 2.664)	7.07
	ωCL	SD, inter-individual CL	0.476	(0.365, 0.587)	11.92
	ωVc	SD, inter-individual Vc	0.535	(0.393, 0.677)	13.56
	ωQ	SD, inter-individual Q	0.468	(0.183, 0.753)	31.08
	ωVp	SD, inter-individual Vp	0.362	(0.249, 0.476)	16.02
	σ (constant)	SE, additive error	0.064	(0.004, 0.125)	48.19
	σ (proportional)	SE, proportional error	0.199	(0.185, 0.213)	3.58
10-1074-LS	F	Bioavailability after SC administration	0.593	(0.518, 0.668)	6.48
	Ka (1/day)	Absorption rate constant	0.294	(0.252, 0.336)	7.31
	CL (L/day)	Clearance from the central compartment	0.026	(0.023, 0.03)	6.74
	Vc (L)	Volume of the central compartment	1.562	(1.308, 1.816)	8.3
	Q (L/day)	Inter-compartmental distribution clearance	0.203	(0.127, 0.279)	19.11
	Vp (L)	Volume of the peripheral compartment	1.334	(1.11, 1.557)	8.54
	ωCL	SD, inter-individual CL	0.341	(0.275, 0.408)	9.92
	ωVc	SD, inter-individual Vc	0.417	(0.322, 0.511)	11.6
	ωQ	SD, inter-individual Q	0.891	(0.625, 1.156)	15.21
	ωVp	SD, inter-individual Vp	0.322	(0.205, 0.44)	18.61
	σ (constant)	SE, additive error	0.342	(0.262, 0.423)	12
	σ (proportional)	SE, proportional error	0.195	(0.184, 0.207)	3.09

Supplemental Table S5: LS/Parental mean value and fold change for each pharmacokinetic (PK) parameter via the targeted minimum loss-based estimation (TMLE) method. mAb, monoclonal antibody; CI, confidence interval, CL, clearance; Vc, central volume; Vp, peripheral volume, AUC, area under the curve.

PK features	mAbs	Parental: Mean (95% CI)	LS: Mean (95% CI)	LS/Parental: Ratio (95% CI)	Two-sided raw p-value	Two-sided adjusted p-value
CL (L/day)	VRC01	0.35 (0.33, 0.37)	0.04 (0.03, 0.04)	0.11 (0.09, 0.12)	<0.001	<0.001
	VRC01/07	0.35 (0.33, 0.37)	0.12 (0.11, 0.12)	0.34 (0.31, 0.36)	<0.001	<0.001
	3BNC117	0.66 (0.59, 0.75)	0.07 (0.06, 0.08)	0.11 (0.09, 0.13)	<0.001	<0.001
	PGDM1400	0.21 (0.2, 0.22)	0.05 (0.05, 0.06)	0.24 (0.21, 0.28)	<0.001	<0.001
	PGT121	0.31 (0.29, 0.34)	0.06 (0.05, 0.07)	0.19 (0.16, 0.22)	<0.001	<0.001
	10-1074	0.16 (0.15, 0.19)	0.03 (0.02, 0.03)	0.16 (0.14, 0.19)	<0.001	<0.001
Vc (L)	VRC01	1.98 (1.83, 2.14)	1.18 (0.92, 1.51)	0.6 (0.47, 0.76)	<0.001	<0.001
	VRC01/07	1.97 (1.84, 2.12)	3.13 (2.73, 3.59)	1.58 (1.37, 1.84)	<0.001	<0.001
	3BNC117	4.25 (3.82, 4.73)	3.13 (2.42, 4.05)	0.74 (0.56, 0.98)	0.033	0.033
	PGDM1400	3.27 (2.86, 3.73)	3.62 (3.28, 3.99)	1.11 (0.96, 1.27)	0.157	0.157
	PGT121	4.18 (3.65, 4.77)	2.76 (2.31, 3.31)	0.66 (0.53, 0.82)	<0.001	<0.001
	10-1074	2.56 (2.27, 2.89)	1.6 (1.39, 1.86)	0.63 (0.52, 0.75)	<0.001	<0.001
Vp (L)	VRC01	4.22 (3.95, 4.51)	1.98 (1.67, 2.36)	0.47 (0.39, 0.56)	<0.001	<0.001
	VRC01/07	4.22 (3.97, 4.48)	3.92 (3.48, 4.43)	0.93 (0.82, 1.05)	0.247	0.247
	3BNC117	9.53 (8.74, 10.41)	2.83 (2.24, 3.58)	0.3 (0.23, 0.38)	<0.001	<0.001
	PGT121	5.19 (4.73, 5.69)	2.96 (2.63, 3.33)	0.57 (0.5, 0.66)	<0.001	<0.001
	10-1074	2.32 (2.03, 2.66)	1.37 (1.07, 1.74)	0.59 (0.45, 0.77)	<0.001	<0.001
Elimination half-life (days)	VRC01	15.14 (14.5, 15.82)	62.47 (58.79, 66.37)	4.13 (3.83, 4.45)	<0.001	<0.001

	VRC01/07	15.13 (14.54, 15.75)	46.57 (44.66, 48.57)	3.08 (2.91, 3.25)	<0.001	<0.001
	3BNC117	16.91 (15.32, 18.67)	65.35 (61.86, 69.03)	3.86 (3.45, 4.34)	<0.001	<0.001
	PGDM1400	21.08 (19.05, 23.33)	55.81 (21.1, 147.64)	2.65 (1, 6.99)	0.049	0.148
	PGT121	24.26 (22.19, 26.52)	70.92 (66.09, 76.1)	2.92 (2.6, 3.28)	<0.001	<0.001
	10-1074	23.17 (21.52, 24.95)	81.42 (75.97, 87.25)	3.51 (3.18, 3.89)	<0.001	<0.001
Dose normalized steady-state AUC	VRC01	2.84 (2.69, 3)	26.91 (24.11, 30.04)	9.46 (8.42, 10.63)	<0.001	<0.001
	VRC01/07	2.85 (2.71, 2.99)	8.47 (8.01, 8.94)	2.97 (2.78, 3.18)	<0.001	<0.001
	3BNC117	1.51 (1.34, 1.7)	13.91 (12.49, 15.48)	9.23 (7.84, 10.86)	<0.001	<0.001
	PGDM1400	4.79 (4.5, 5.09)	19.7 (17.6, 22.05)	4.11 (3.62, 4.68)	<0.001	<0.001
	PGT121	3.19 (2.94, 3.46)	16.82 (15.15, 18.69)	5.27 (4.56, 6.1)	<0.001	<0.001
	10-1074	6.07 (5.39, 6.84)	37.35 (32.85, 42.47)	6.15 (5.22, 7.24)	<0.001	<0.001
Predicted concentrations at 4 weeks post 1.4g IV infusion (mcg/ml)	VRC01	39.05 (36.59, 41.67)	297.67 (266.39, 332.64)	7.62 (6.76, 8.6)	<0.001	<0.001
	VRC01/07	39.1 (36.83, 41.5)	104.99 (99.01, 111.34)	2.69 (2.49, 2.89)	<0.001	<0.001
	3BNC117	22.16 (19.39, 25.32)	145.82 (130.22, 163.29)	6.58 (5.55, 7.8)	<0.001	<0.001
	PGDM1400	71.78 (66.43, 77.56)	240.12 (219.25, 262.97)	3.35 (2.99, 3.74)	<0.001	<0.001
	PGT121	48.34 (44.31, 52.73)	169.23 (152.67, 187.59)	3.5 (3.02, 4.06)	<0.001	<0.001
	10-1074	96.53 (85.68, 108.74)	345.37 (304.63, 391.56)	3.58 (3.05, 4.19)	<0.001	<0.001

Supplementary Figure S1: Serum concentrations (mcg/mL) for each monoclonal antibody pooled across clinical trials. Lines depict trajectory for an individual participant with color denoting administration type (IV, intravenous; SC, subcutaneous).

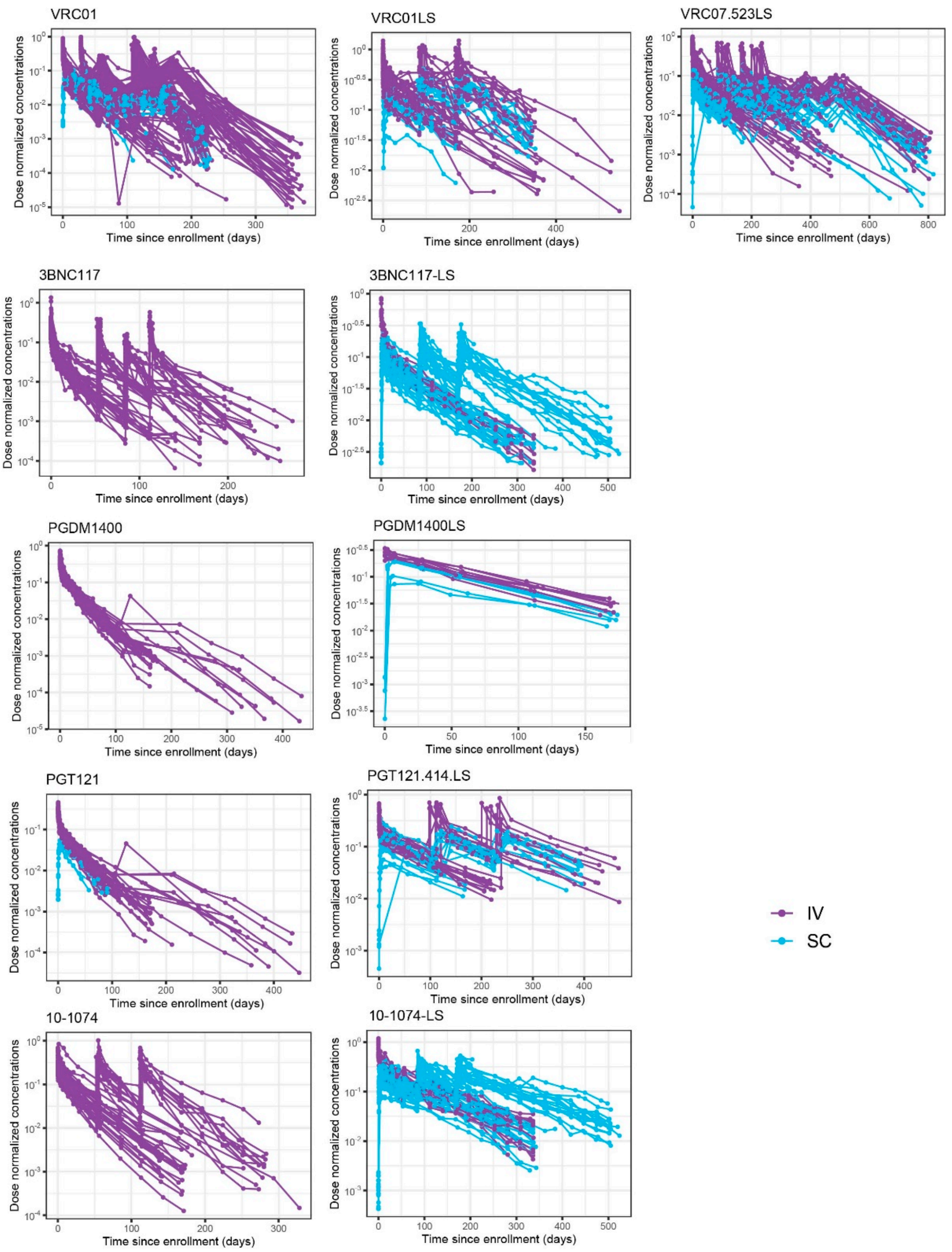

Supplemental Figure S2: Individual-level predictions for each pharmacokinetic (PK) parameters comparing models fit to clinical trials separately vs. combined (pooled. CL, clearance; Vc, central volume; Vp, peripheral volume; AUC, area under the curve; IV, intravenous; SC, subcutaneous.

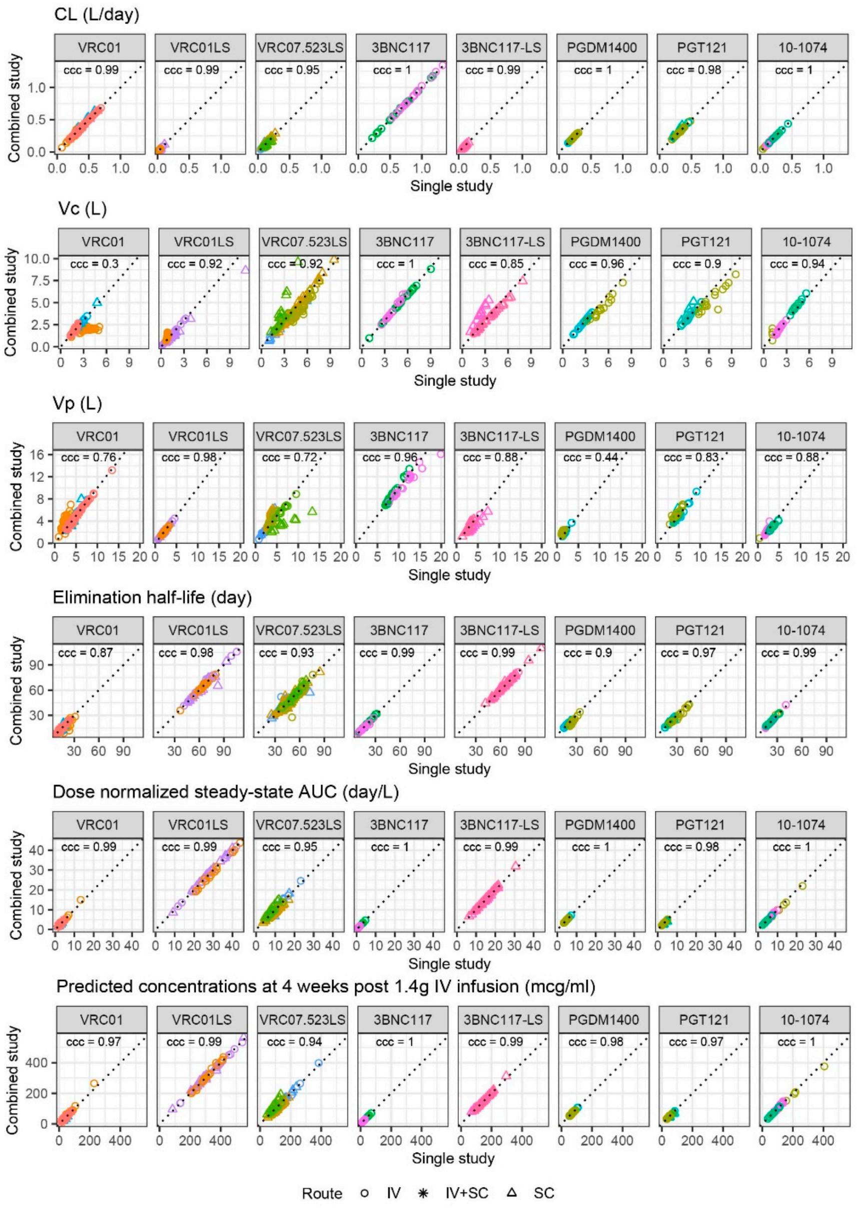

Supplemental Figure S3: Diagnostic plots of all pharmacokinetic models pooled over monoclonal antibodies.

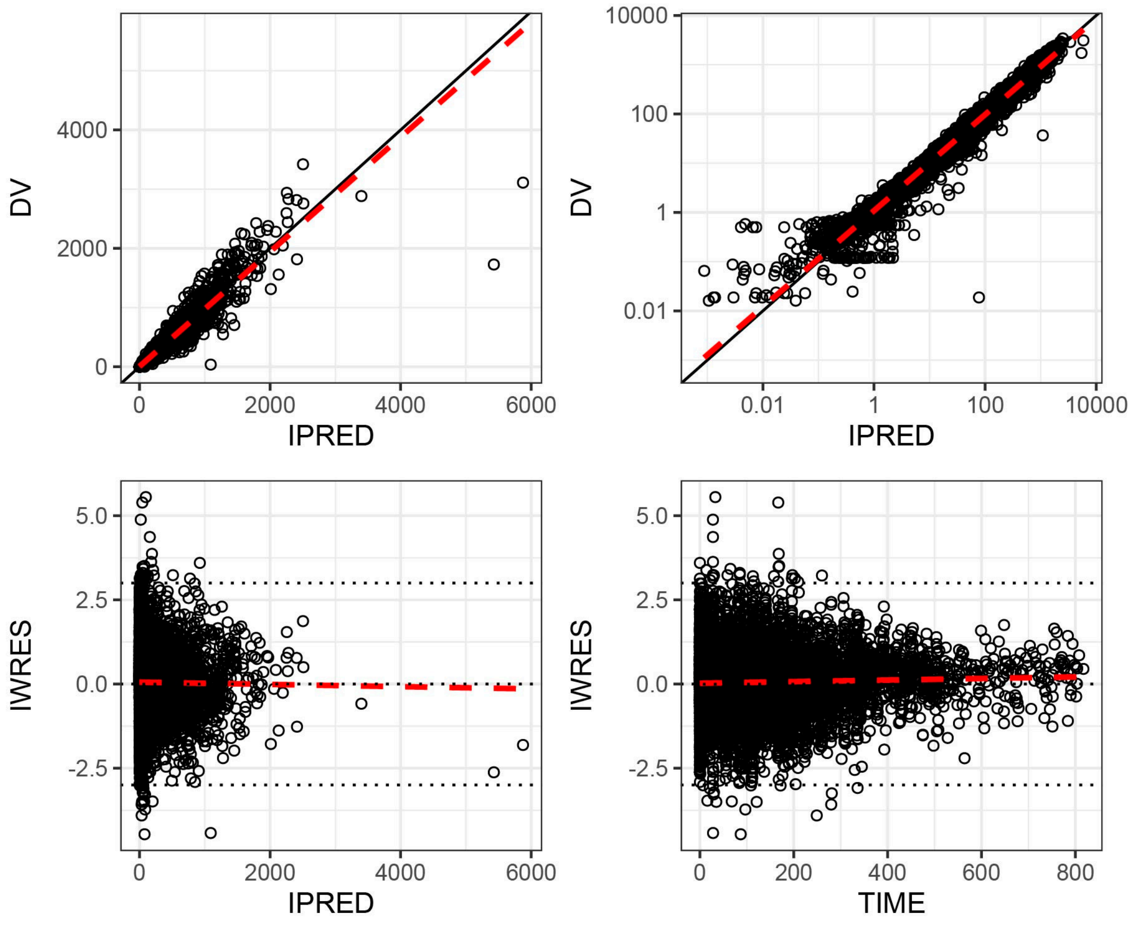

Supplemental Figure S4: Scatter visual predictive check (VPC) plot for each mAb by subcutaneous (SV and intravenous (IV administration. Lines depict the median trajectory, shaded region depicts 90% prediction interval, and the points depict individual participant observation (DV). Values are dose normalized for comparison.

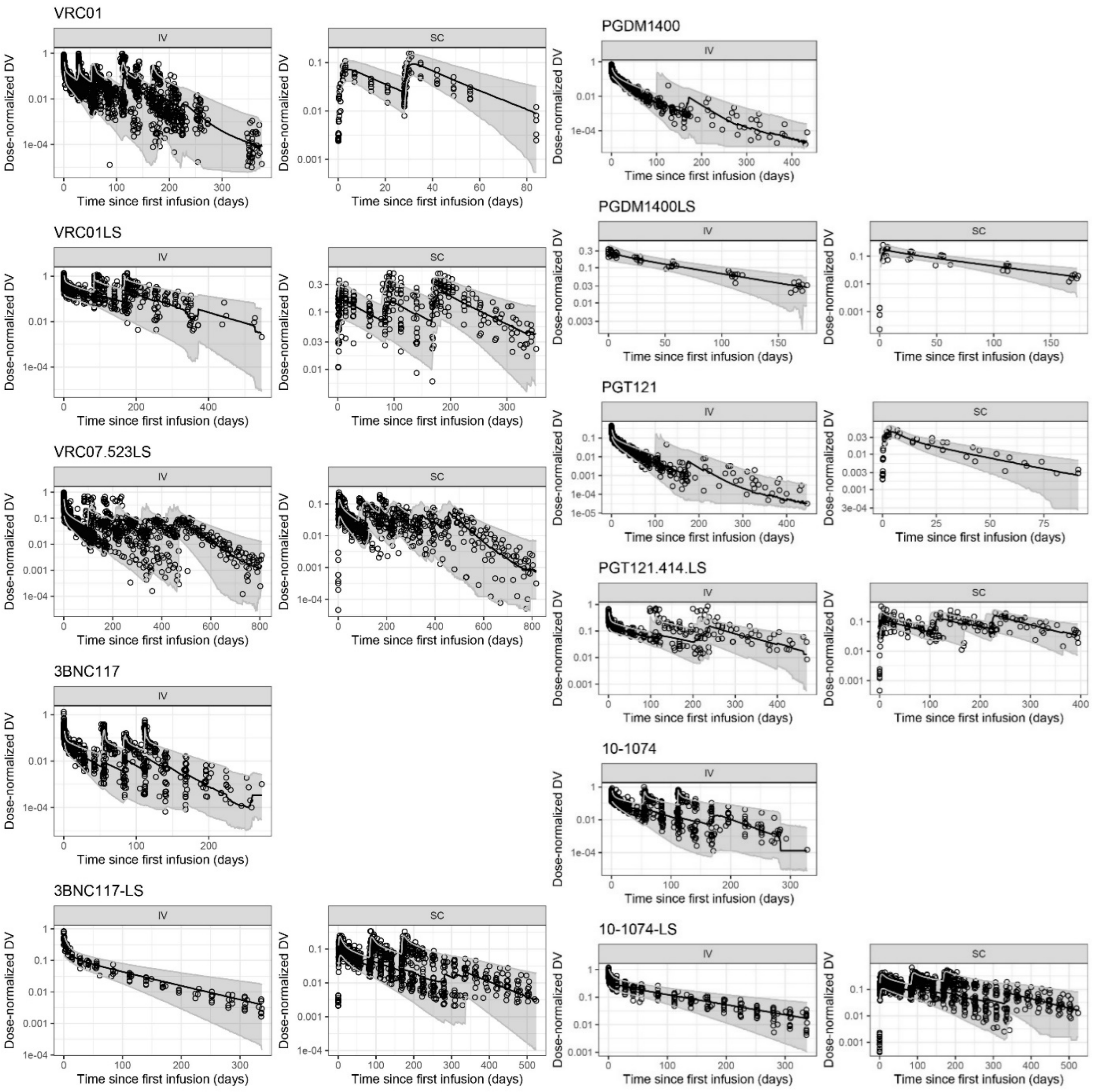

Supplemental Figure S5: Scatter visual predictive check (VPC) plot for each monoclonal antibody pooled over administration type.

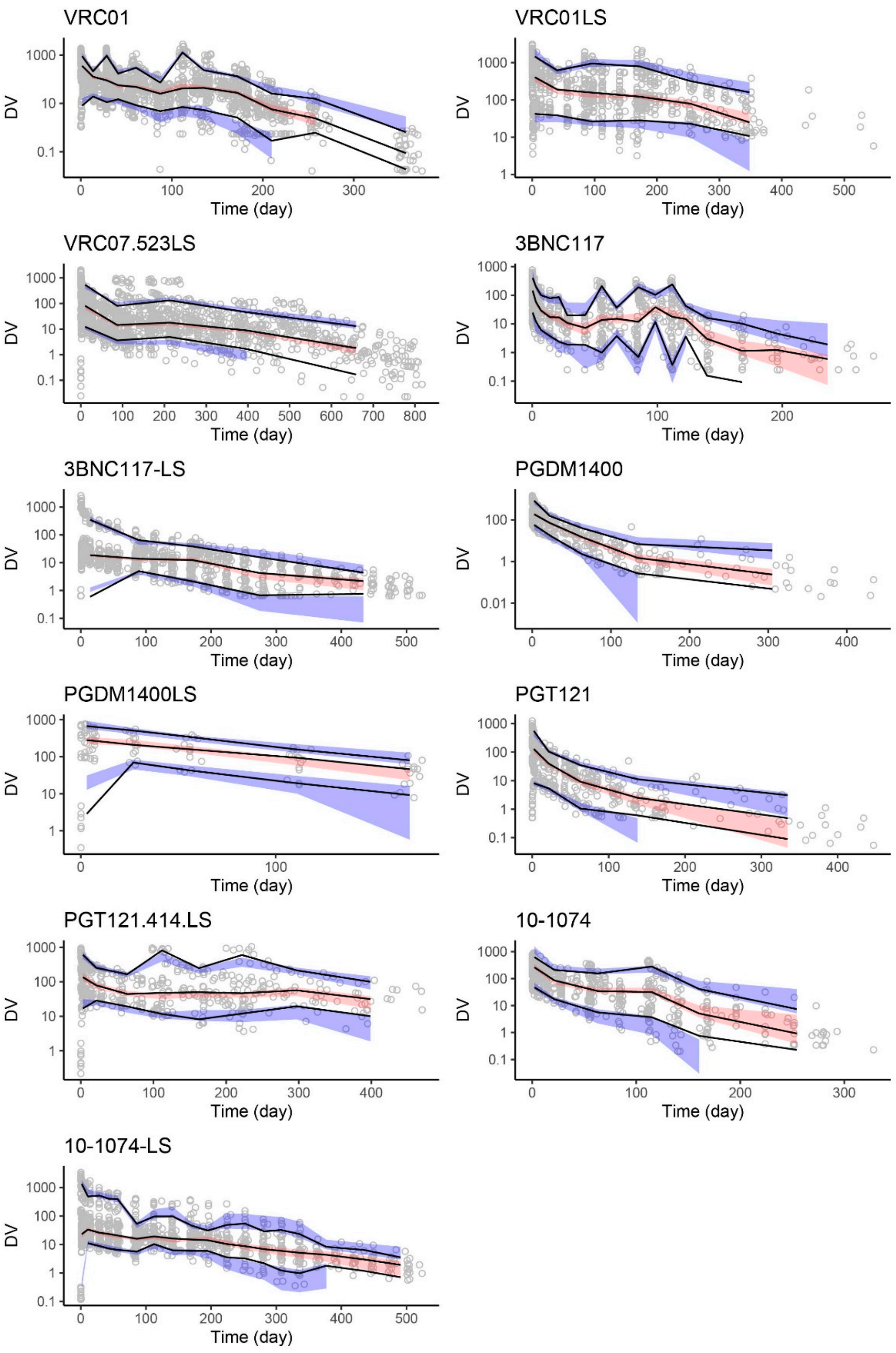

Supplement: Supplementary file 1 [file pharmaceutics-16-00594-s001.zip › pharmaceutics-2985042-Supplemental Materials.pdf]
